# Gut microbiota, epigenetic modification and colorectal cancer

**Published:** 2017-04

**Authors:** Sama Rezasoltani, Hamid Asadzadeh-Aghdaei, Ehsan Nazemalhosseini-Mojarad, Hossein Dabiri, Reza Ghanbari, Mohammad Reza Zali

**Affiliations:** 1 Basic and Molecular Epidemiology of Gastrointestinal Disorders Research Center, Research Institute for Gastroenterology and Liver Diseases, Shahid Beheshti University of Medical Sciences, Tehran, Iran; 2 Gastroenterology and Liver Diseases Research Center, Research Institute for Gastroenterology and Liver Diseases, Shahid Beheshti University of Medical Sciences, Tehran, Iran; 3 Department of Medical Microbiology, School of Medicine, Shahid Beheshti University of Medical Sciences, Tehran, Iran; 4 Digestive Oncology Research Center, Digestive Diseases Research Institute, Tehran University of Medical Sciences, Tehran, Iran

**Keywords:** Gut microbiota, Colorectal cancer, Epigenetic modification

## Abstract

Micro-organisms contain 90% of cells in human body and trillions foreign genes versus less than 30 thousand of their own. The human colon host various species of microorganisms, appraised at more than 10^14^ microbiota and contained of over a thousand species. Although each one’s profile is separable, the relative abundance and distribution of bacterial species is the same between healthy ones, causing conservation of each person’s overall health. Germline DNA mutations have been attributed to the less than 5% of CRC occurrence while more than 90% is associated with the epigenetic regulation. The most ubiquitous environmental factor in epigenetic modification is gut microbiota. Disruptive changes in the gut microbiome strongly contributed to the improvement of colorectal cancer. Gut microbiota may play critical role in progression of CRC via their metabolite or their structural component interacting with host intestinal epithelial cell (IEC). Herein we discuss the mechanism of epigenetic modification and its implication in CRC development, progression even metastasis by gut microbiota induction.

## INTRODUCTION

### Microbiota and types of disease.

The human intestinal tract contains various species of microorganisms, about 10^14^ bacteria and contained of over a thousand species. Each person’s gut microbial profile influenced by a number of factors including but not limited to age, sex, genetics, diet, and lifestyle. Although each individual’s profile is separable, the relative abundance and distribution of bacterial species is similar among healthy ones, resulting in each person’s overall health establishment (1, 2). The relationship between gut flora and human is a mutualistic relationship. Gut microbiota provide a host of useful functions, in return, the host intestine provides nutrient rich environment in which microbiota can thrive and aid the host homeostasis modulation (3). Hence, the dysbiosis of healthy gut microbiota may correlate to the deterioration of host and microbiota mutualistic relationship leading to the several disorders (4).

Changes of gut microbiota has been linked to many diseases such as neuropsychological disease including depression (5), stress (6) and autism spectrum disorder (7), metabolic disorders such as obesity (8), metabolic syndrome (9), atherosclerosis (10), type 1 Diabetes (11), type 2 Diabetes (12) and gastrointestinal disorders including Crohn’s disease (13), inflammatory bowel disease (14), irritable bowel syndrome (15), colorectal cancer (16), ulcerative colitis (17), liver disease (18) and nonalcoholic fatty liver disease (19). Other diseases which are related to the gut microbiota include auto immune disorder (20), allergy (21), cardiac asthma (22), Alzheimer (23), cardiovascular disease (24), and celiac (25). Fortunately, studies have also demonstrated that gut microbiota may be modulated with the use of prebiotics, probiotics, antibiotics, and fecal transplantation of microbiota as gut flora associated diseases therapeutic agents. This modulation of gut microbiota is currently an important area of research as it just might find the solution for related diseases treatment (26). Gaining a wide view of the microbial environment in our gut-microbiome has become possible with high-throughput micro environmental sequencing techniques. Many medium-scale studies have been newly identified the microbiota of colonic tumor biopsies compared to healthy mucosa with metatranscriptomic sequencing or quantifying the 16S rRNA phylogenetic marker gene (27).

### The gut microbiota community.

Several studies have been identified that the health of gut microbiome is directly related to the health of the host. Intestinal gut microbiome ecosystem can be considered the largest endocrine organ in the body which enables to produce various metabolite products and biologically active composition like hormones, that may be circulated and distributed to different body sites of the host, and affected diverse vital biological processes. Gut microbiome ecosystem fulfilled all the requirements for description as an endocrine organ because it is plastic and generating distinct biologically active component (28).

For example the human gut is defined as reservoir for the lipopolysaccharide (LPS) of Gram negative bacteria which is the major composition of their outer membrane. Many studies have described that low rate of LPS are acquirable in the blood of healthy individuals proposing that LPS is continuously absorbed at a low level from the gut (8).

For consecutive years, studying of enteropathogenic bacteria through the field of host/bacterial interaction was pioneering and great efforts have been complied to understand their precise molecular mechanisms of pathogenicity. However the enteropathogenic bacteria present a small proportion (3%) of the total microbial community in GI tract and above all, the most of these microorganisms not only are not natural residents but also they are transient and rarely impact long term effects (29).

As long as more than 10^14^ microorganism present in the GI tract and most of them attendance in the colon, researchers have made a thoughtful question about their potency in health and disease. This field of studies is recent one and worldwide efforts are underway to identify human microbiome (30).

With using next generation sequencing and pyrosequencing techniques based on ribosomal 16S bacterial genes, a new insight opened to the identification of intestinal gut microbiota (29). Despite the use of these techniques, the gut microbiome composition has not been definitely identified (29, 30). However there is an estimation that gasterointestinal tract at the phylum level is predominated colonized by the Firmicutes (∼75%) and Bacteroidetes (∼20%) followed by the Proteobacteria and Actinobacteria.

Currently researchers have identified various microbial groups associated with CRC. Although these studies have not proved a unique group of bacteria correlated with CRC, these findings have generally demonstrated differences between healthy and disease gut microbiome. For instance Sobhani et al. in 2011 reported higher population of *Bacteroides* and *Prevotella* group in CRC patient compared with healthy ones (30). Wang et al. in 2011 proved the genera *Enterococcus, Escherichia/Shigella, Klebsiella, Streptococcus,* and *Peptostreptococcus* were significantly increased in CRC patients than healthy individuals, while Lachnospiraceae family which produce short chain fatty acid particularly butyrate were less abundant (31). An increased abundance of *Fusobacterium* in rectal swab samples in patients with CRC compared with healthy group was also described by other studies (32, 33). Sanapareddy et al. in 2012 identified an expansion of Firmicutes, Bacteroidetes, and Proteobacteria in the intestinal mucosal surface of adenomatous patient compared with non-adenomatous cases (34). Also Chen et al. in 2012 identified increases in *Coriobacteridae, Roseburia, Fusobacterium,* and *Faecalibacterium* genera while Enterobacteriaceae family decreased (35).

Herein it is discussed the mechanism of epigenetic modification and its implication in CRC development, progression even metastasis by gut microbiota induction.

#### Fusobacterium nucleatum.

*F. nucleatum* invasion and carcinogenesis has been explained by the FadA activated complex adhesion. The FadA links to the extracellular E cadherin and induces activation of B catenin/Wnt signaling via cell proliferation and tumor development stimulation (36). *F. nucleatum* expands myeloid derived immune cells in tumor environment and mediates inflammatory responses. This bacterium is an asaccharolytic, so versus the Enterobacteriaceae, *F. nucleatum* is unwilling to take glucose which is substrate for tumor development. Instead the bacterium consumes peptides and amino acids in tumor microenvironment and produces amino acid metabolite such as methionyl, formyl, phenylalanine, leucyl, and short-chain fatty acids. Moreover, *F. nucleatum* is able to resist in hypoxic tumor microenvironment and even slowly replicate. Unlike several strict anaerobes this bacterium owns a primary electron transport chain, with little capability to respire oxygen (37, 38).

#### Streptococcus gallolyticus.

*S. gallolyticus* or former *S. bovis* biotype I is a nonenterococcal group D *Streptococcus*, which is considered as normal gut microflora of the human in 5% to 16% of adults. Currently it is identified that the bacterium has a potential role in the increase of colorectal neoplasia risk.

The bacterium releases a pilus protein with a collagen binding domain (coded by the pil1 locus) which enhances inflammation and causes growth priority under metabolic position. Moreover, *S. gallolyticus* proteins are contributed to the overexpress of cyclo-oxygenase-2 (Ptgs2) which is often increased in the colon cancer and prevents apoptosis and promotes angiogenesis in an *in vitro* condition (39, 40).

#### Enterococcus faecalis.

*E. faecalis* strains have different capacity to generate reactive oxygen species inducing DNA damage and chromosomal instability. Above all, *E. faecalis* strains capable to produce extracellular superoxide anions as primer and growth factor of CRC. Carcinogenesis of *E. faecalis* is identified by mucosal macrophages induction for producing diffusible clastogens (chromosomal-breaking factors) such as 4-hydroxy-2-nonenal (a breakdown product of u-6 polyunsatured fatty acids) which regulates DNA damage by bystander effect (41, 42).

#### Enterotoxogenic Bacteroides fragilis.

*Enterotoxogenic Bacteroides fragilis* (ETBF) was identified as a potential microbial motivator of human CRC occurrence based on it’s the only recognized virulence factor, the *B. fragilis* toxin (BFT). The ETBF causes colitis, colonic hyperplasia and tumor initiation in Min mice by signal transducer and activator of transcription 3 (STAT3) and a pro-inflammatory Th17 response (43). BFT is zinc dependent metalloprotease toxin which cleavages tumor suppressor protein, E-cadherin, leading to enhance nuclear Wnt/b-catenin signaling that results in enhanced colonic carcinoma cell proliferation and MYC proto-oncogene expression. Moreover BFT induces NF-kB signaling that promotes colon epithelial cell (CEC) secretion of cytokines which likely associate with improvement of mucosal inflammation; subsequent NF-kB signaling may associate with CEC carcinogenesis mucosal IL17 induction. Also ETBF in vivo and BFT *in vitro* induce DNA damage in CECs (44).

#### Escherichia coli.

Intracellular *E. coli* could be obtained from colorectal tumor. Furthermore genotoxic *E. coli*, and tightly adherent *E. coli* are two groups of *E. coli* which associate with CRC pathogenesis. Between potential genotoxic *E. coli*, phylogenetic group B2 *E. coli* causes double strand DNA breaks through the polyketide synthase (pks) island containing the genotoxincolibactin (45).

#### Helicobacter pylori.

Many cross sectional studies evaluating the potential relationship between CRC and *H. pylori* which has strongly documented higher prevalence of colorectal adenoma and advanced adenoma in the *H. pylori+* than in the *H. pylori-*subjects (46).

### Colorectal cancer and molecular features.

CRC mechanisms are divided into three categories: genetic, epigenetic and aberrant immunologic signaling pathway (47). These pathways may be categorized on the basis of three molecular features (48, 49): (i) Mutations in DNA mismatch repair genes, causing DNA microsatellite instability (MSI) phenotype (50) which could be correlated with some gut bacterial induction like genotoxic *E. coli* and tightly adherent *E. coli* (45). (ii) Mutations in *APC* and those genes that activate Wnt pathway, determined by chromosomal instability (CIN) phenotype (51–54). Some strains of bacteria have four different capacity to produce reactive oxygen species enable DNA damage induction and chromosomal instability like *Enterococcus faecalis* (41, 42). (iii) Global genome hypermethylation leads to switch off tumor suppressor genes, explained as CpG island methylator phenotype (CIMP) (55). Some bacterial genus like Flavinofractor inversely has been correlated with the methylation of genes while some of others like *Peptostreptococcus* and *Schwartzia* genus directly have been correlated with colorectal carcinogenesis related gene methylation (65).

More than 90% of CRC occurrence is associated with the epigenetic regulation while germline DNA mutations have been attributed to the less than 5% of patients. Epigenetic mechanisms of CRC includes: miRNA regulation, DNA methylation, histone modification such as acetylation, methylation, phosphorylation and ubiquitylation (47).

### Gut microbiota and epigenetic modification.

The relationship between cancer and epigenetic modification returns to 1983 (56). With innovation of various new techniques to study epigenetic mechanism in gene expression modulation, epigenetics has become popular area of research in the field of cancer biology even cancer therapy (47). As it discussed above, human microbiome take a part in human physiology and may also be responsible for genome complexity mystery. It would be interesting to give an example: why the 26, 600 protein encoding transcriptosome Homo sapiens are much fewer in number than for instance the rice genome (Oryzasativa; which has about 46,000 functional genes)?

Many different species and strains of bacteria may participate up to 4 × 10 ^6^ potential mRNAs to the human transcriptome, so making the human host plus microbiome genetic complex it closer to 4, 026, 600 mRNA transcripts, and obvious “winner” of human genetic complexity over that of rice and other species (57–59).

Microbes have an essential role in the biological microenvironment: about 16% of cancers have been recognized to be caused by microbes and those related to the liver and gastrointestinal tract are clearly identified as being microbe related (60). For example *H. pylori* is contributed to the adenocarcinoma and MALT lymphoma (61).

Epigenetic regulation of oncogenes, proinflammatory mediators, tumor suppressor and miss match repair genes identify as significant mechanism by which homeostatic balance is lost and dysbiosis phenomenon is occurred (47).

Two phyla; Bacteroidetes and Firmicutes that are belonging to the obligate anaerobic bacteria were significantly more prevalent in the luminal compartment where more than 50 different phyla inhabit. Other phyla, consist of the Proteobacteria, Verrucomicrobia, and Actinobacteria, are present far fewer in number. Dysbiosis of this composition is characterized by a considerable decrease in the resident obligate anaerobic bacteria; whereas facultative anaerobes such as Enterobacteriaceae increase. Appropriate and balanced gut microbiome plays a vital role in the development and maturation of a healthy immune system (62, 63).

Gut microbiota may play critical role in progression of CRC via their metabolite or their structural component which interacting with pathogen-associated molecular patterns (PAMP) and microbe-associated molecular pattern (MAMP) receptors such as Toll-like receptors (TLRs). TLRs have critical role in microbe recognition, innate immunity and maintaining homeostasis in the intestinal microenvironment. In the healthy gut, TLR3 and TLR5 seem to be ongoing expressed. Versus, TLR2 and TLR4 are expressed at very low levels, resulting that this regulation is critical to prevent autoinflammatory immune activation in response to commensal microbes (64).

### MicroRNas.

MicroRNAs (miRNAs) are small (18-25nt) non-coding RNAs that regulate target genes translation by degradation of mRNA induction or translating inhibition. It has been shown that the miRNAs can be utilized as markers for different types of disease (65). Currently miRNA utilization provides appropriate chance for early identification of cancers. Changes in miRNAs expression have been observed in several types of cancer, particularly CRC. Differential expressions of the miRNAs in serum, plasma, and stool of patients with CRC have also been observed (65–67). For instance, the expression of miR-4478 and miR-1295b-3p were strongly reduced in stool samples of CRC patient with early stage (I and II) in comparison with healthy individuals (68).

It is also demonstrated that the expression levels of plasma miR-142-3p and miR-26a-5p were significantly diminished in patients with colorectal cancer in comparison with normal subjects (69).

Seven miRNAs were found to be under expressed in both plasma and stool samples of patients with CRC compared with healthy individuals. The miRNAs may be appropriate candidate as noninvasive diagnostic molecular and prognostic biomarkers for CRC because of their small size and their stability in various biological samples; as far as researchers pointed extracellular miRNAs are stable for at least 1 month, even in stool samples (70).

Currently experiments have been indicated that gut microflora derived miRNA and small non coding RNA (sncRNA) that signal between cells, tissues and also may be between individual species demonstrated that human being might be considerably influenced by the gut microbiome function regulated miRNA and sncRNA trafficking (71, 72).

### DNA methylation.

DNA methylation is an important epigenetic modifications which is the first global epigenetic mechanism identified in cancer initiation and progression. In this way DNA methyltransferase (DNMT) enzymes add a methyl group (CH3) to carbon 5 of the cytosine base, usually in CpG dinucleotides. Aberrantly hyper methylated CpG islands may lead inappropriate silencing of gene expression. Aberrant genomic methylation is considered to result in tumorigenesis by deregulating gene expression of key genes (73). For instance DNA mismatch repair genes such as MGMT, MLH1 and also tumor suppressor genes could be silenced by hypermethylation (74). The RUNX3 which regulates both Wnt/b-catenin signaling pathways and transforming growth factor (TGF)-b, could be hypermethylated and perhaps lead to the gastric epithelial cell proliferation and diminish apoptosis and reduce sensitivity to the growth inhibitory cytokine (75, 76). APC CPG region promoter hypermethylation can cause CRC with overexpression of CMYC and the oncogenes which is a downstream effector of the Wnt/b-catenin pathway. Wide range of genome hypomethylation has been identified in several human cancers (77). Hypermethylation results in inactivation of tumor suppressor genes in CRC, while global genomic hypomethylation performs a critical role in tumor foundation via proto oncogenes activating or by inducing chromosomal instability. With genes lacking CpG-rich promoter regions, hypomethylation of the gene body may attribute to the cancer initiation and progression (78).

In recent studies the interplay between gene methylation and gut microbiome in CRC has been identified. For example in one study *Flavinofractor* genus inversely correlated to methylation of genes occurring at the first steps of the colorectal carcinogenesis while *Peptostreptococcus* and *Schwartzia* genus directly correlated with colorectal carcinogenesis related gene methylation (79). In other study, the Firmicutes phyla directly correlated and *Bacteroides* group inversely correlated with CDH13 methylation (80).

### Histone modification.

Although the vast researches aimed at intending DNA methylation in CRC development, far fewer is known about the potential association of aberrant histone post translational modifications to the CRC imitation and progression even metastasis (81).

The relationship between histone deacetylation and DNA methylation in cancer initiation was reported previously. Experimental evidence had reported that those genes with hyper methylated at their promoter CpG islands are usually contributed with histone deacetylation.

The unit of chromatin is nucleosome, that consists of histone octamer (two copies each of the four core histone proteins H2A, H2B, H3, and H4) and 146 base pairs of DNA are wrapped around that (81, 82).

Post translational methylation of the N terminal of histones may have adverse roles in the expression of target genes. Unfavorable methylation of histone, histone acetylation tails by histone acetyltransferases (HATs) strongly create active site. The Acetyl group is added to lysine to neutralize the positive histone charge. Disruption between neutral histone charge and negatively charged DNA leads to the higher open chromatin structure for more availability of DNA to transcription factors (83–85). The histone deacetylases (HDACs) are a class of enzymes which omit acetyl groups from acetylated histones, reversing the open chromatin structure, and leading a condensed heterochromatic position and also inactivation of transcription. Pursuantly, cooperation of HATs and HDACs results in maintaining the balance of histone acetylation *in vivo* to establish homeostasis (85). Experimental evidences have shown that bacteria directly influence DNA replication, transcription, repair system, RNA splicing, and chromatin remodeling (83–85).

## CONCLUSION

It is concluded that wider view of human metabolism, disease and physiology must be attended. Actually human microbiome takes a part in human physiology and may also be responsible for genome complexity mystery. The mutualistic relationship among host and gut microbiota offers advantages to host by several ways. Any disorder in the gut microbiome balance will alter microbial composition and also initiate aberrant intestinal signaling pathways and epi-genetic modification. In addition bacterial metabolic end-products and their surface toxins may induct strong inflammation which can leads to malignancy due to dysbiosis. Any way we are walking societies consisted not only of a Homo sapiens host, but also of trillions of symbiotic commensal microorganisms on every surface of our bodies particularly gut.
